# Response Advantage for the Identification of Speech Sounds

**DOI:** 10.3389/fpsyg.2020.01155

**Published:** 2020-06-12

**Authors:** Howard S. Moskowitz, Wei Wei Lee, Elyse S. Sussman

**Affiliations:** ^1^Department of Otorhinolaryngology-Head and Neck Surgery, Albert Einstein College of Medicine, New York, NY, United States; ^2^Department of Neuroscience, Albert Einstein College of Medicine, New York, NY, United States

**Keywords:** speech detection, sound category, switch trials, repeat trials, identification

## Abstract

The ability to distinguish among different types of sounds in the environment and to identify sound sources is a fundamental skill of the auditory system. This study tested responses to sounds by stimulus category (speech, music, and environmental) in adults with normal hearing to determine under what task conditions there was a processing advantage for speech. We hypothesized that speech sounds would be processed faster and more accurately than non-speech sounds under specific listening conditions and different behavioral goals. Thus, we used three different task conditions allowing us to compare detection and identification of sound categories in an auditory oddball paradigm and in a repetition-switch category paradigm. We found that response time and accuracy were modulated by the specific task demands. The sound category itself had no effect on sound detection outcomes but had a pronounced effect on sound identification. Faster and more accurate responses to speech were found only when identifying sounds. We demonstrate a speech processing “advantage” when identifying the sound category among non-categorical sounds and when detecting and identifying among categorical sounds. Thus, overall, our results are consistent with a theory of speech processing that relies on specialized systems distinct from music and other environmental sounds.

## Introduction

How we extract meaningful information from the auditory signal is still not fully understood. An important auditory skill is the ability to distinguish among the different types of sounds in the environment and to identify sound sources such as a car honking, a person talking, or music playing. Classifying an auditory object involves distinguishing the various characteristics of the sound, such as its pitch, envelope, and rhythm. The listener may not recognize a specific object from which sound emanates (e.g., flute or person) but may still be able to classify the category of sound it belongs to (e.g., music or speech). Previous studies have confirmed that sound categories can be readily differentiated from each other with only limited information (e.g., with only 20–50 ms sound duration) (Murray et al., 2006; Bigand et al., 2011; Agus et al., 2012; Suied et al., 2014; Ogg et al., 2017), but there is some controversy as to whether processes used to identify speech sounds differ from those used to identify other environmental sounds (Murray et al., 2006; Bigand et al., 2011; Agus et al., 2012).

There is a long history of investigation into the distinctiveness of speech perception over other environmental sounds (Eimas, 1974; Pisoni and Lazurus, 1974; Pisoni, 1979; Liberman and Mattingly, 1989; c.f., Krentz and Corina, 2008). Speech sounds have been considered to be specialized for processing, with evidence from infancy suggesting that speech perception is special (Eimas, 1974; Pisoni, 1979; Vouloumanos and Werker, 2007; Gervain and Geffen 2019), and in childhood results show that speech sounds distract differently than nonlinguistic sounds (Cherry, 1981). However, it is not clear what type of processing “advantage,” if any, exists in adulthood. Recent functional magnetic imaging work has conferred with earlier studies. These studies demonstrate a specialization of sound processing in the brain that designates different pathways for speech and music sounds (Vouloumanos et al., 2001; Kriegstein and Giraud, 2004, Lewis et al., 2004, 2005; Chartrand et al., 2008; Petkov et al., 2008; Norman-Haignere et al., 2015), and shows that the brain processes speech faster than nonlinguistic sounds (Parviainen et al., 2005), thus potentially affording an advantage for speech processing.

Previous studies reporting a speech advantage have primarily studied the speed of detection by comparing speech to other sounds in go/no-go tasks (Murray et al., 2006; Agus et al., 2010). For example, in Agus et al. (2010), voice stimuli were designated as targets (go response) and music designated as distractors (no-go response) and the reaction time when speech was the target compared to when music was the target showed a faster response time to voices than music stimuli. In other studies, the goal was to determine how brief a stimulus could be and still obtain accurate categorical responses to speech or other categories of sounds (Ballas, 1993; Murray et al., 2006; Bigand et al., 2011; Agus et al., 2012; Ogg et al., 2017). These studies focused more on differences in the acoustic characteristics of the sounds and did not generally differentiate other processing factors that could influence response speed and accuracy. For example, previous studies focused on speech compared to non-speech sounds but did not contrast differences that task demands may have on processing time and accuracy when asked to detect or identify categories of sounds. In the current study, we compared response times and response accuracy when detecting the presence of speech compared to when identifying and categorizing the sounds as speech, music, or environmental. In addition, we compared participant expectation as an independent variable that could potentially influence performance. The goal of the current study was to determine if there is a general processing advantage for speech sounds over other familiar environmental sounds; that is, regardless of task, speech sounds are always processed differently than nonlinguistic sounds, or if a speech advantage is conferred only under specific listening or task situations. Thus, we compared responses to speech, music, and environmental sounds in different stimulus contextual conditions and with different task demands to test the hypothesis that speech is processed faster and more accurately than non-speech.

To test our hypothesis, we differentiated detection and identification by using the same categorical sounds in three different task paradigms in which target responses were triggered by either unexpected or expected categories of sounds. In two of the conditions, an oddball paradigm with a random distribution of stimuli was used in which the target categorical stimuli occurred infrequently among nonlinguistic complex tones. Targets were to be detected but not identified (*Detection* condition) or detected and identified (*Identification* condition). In the third condition, only categorical sounds were presented in a predictable pattern. Thus, the sound category (speech, music, or environment) could be implicitly derived from the repetition of the stimuli. The participant’s task was to identify the sound category of each stimulus with a button press, while each stimulus pattern repeated or switched category (*Repetition-Switch* condition). Thus, identification was always required at the first tone of the pattern in this condition (where it repeated or switched from the previous category) and only detection of the remaining tones of the pattern would be required after the category was identified. With these three conditions, we compared response outcomes for detection and identification when the sound categories were unexpected (*Detection* and *Identification* conditions) and expected (*Repetition-Switch* condition) to determine if expectancy played an additional role in the efficiency of speech perception.

## Materials and Methods

### Participants

Twenty-two adults ranging in age from 21 to 40 years (M = 28, *SD* = 5) were paid to participate in the study. The protocol was approved by the Internal Review Board of the Albert Einstein College of Medicine (Bronx, NY), where the study was conducted. Prior to testing, all participants gave written consent after the protocol was explained to them, in accordance with the Declaration of Helsinki. All participants passed a hearing screening at 20 dB HL at 500, 1,000, 2,000, and 4,000 Hz in the left and right ears and had no reported history of neurological disorders.

A power analysis was conducted using Statistica software to test the difference between two dependent means, using a two-tailed test, a medium effect size (*d* = 0.50), and an alpha of 0.05. Results showed that a total sample of 16 participants was required to achieve a power of 0.90. The 22 participants included in the current study exceed the number required to obtain sufficient statistical power.

### Stimuli

**Figure 1:**
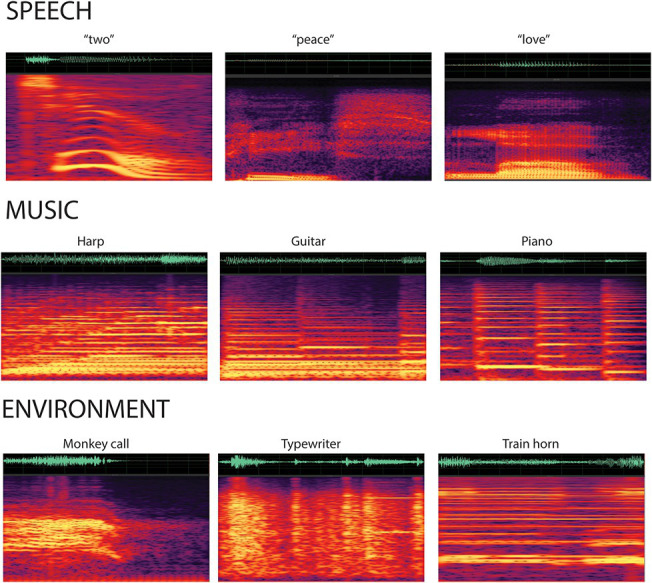
Spectrogram of the categorical sounds. A sample of the sounds from each stimulus category displays the waveforms and spectrograms for the speech (top row), music (second row), and environmental (third row) sounds. Categorical sounds were novel (i.e., did not repeat) within each condition.

Stimuli were complex categorical sounds (32-bit stereo; 44,100 Hz digitization): 25 novel tokens of spoken speech, 25 novel tokens of music, and 57 novel tokens of environmental sounds (Figure 1). Sounds were obtained from free online libraries of sounds: speech sounds were words (e.g., “hello” and “goodbye”); music sounds were taken from various musical instruments (e.g., piano, flute, and bass); and environmental sounds were taken from a range of sources, including nature (e.g., water dripping), vehicles (e.g., engine revving), household (e.g., phone ring), and animals (e.g., bird chirp). All of the sounds were then modified using Adobe Audition software (Adobe Systems, San Jose, CA) to be 500 ms in duration, with an envelope rise and fall times of 7.5 ms at onset and offset to minimize clicks. Half-second samples were sufficient to identify the source of the non-speech sounds (e.g., instrument, bird, and vehicle) that would distinguish categories. The final set of 107 sounds were verified as belonging to a category of speech, music, or environmental by three lab members who were not included in the study. One complex tone (1,000 Hz fundamental frequency with four partials) with 500 ms duration (7.5 ms rise/fall time) was created with Adobe Audition. All 107 stimuli were equated for loudness using the root mean square (RMS) amplitude with Adobe Audition software. Sounds (categorical sounds and the complex tone) were calibrated with a sound pressure level meter in free field (Brüel and Kajaer; Denmark) and presented through speakers at 65 dB SPL with a stimulus onset asynchrony (SOA) of 1.1 s.

### Procedures

**Figure 2:**
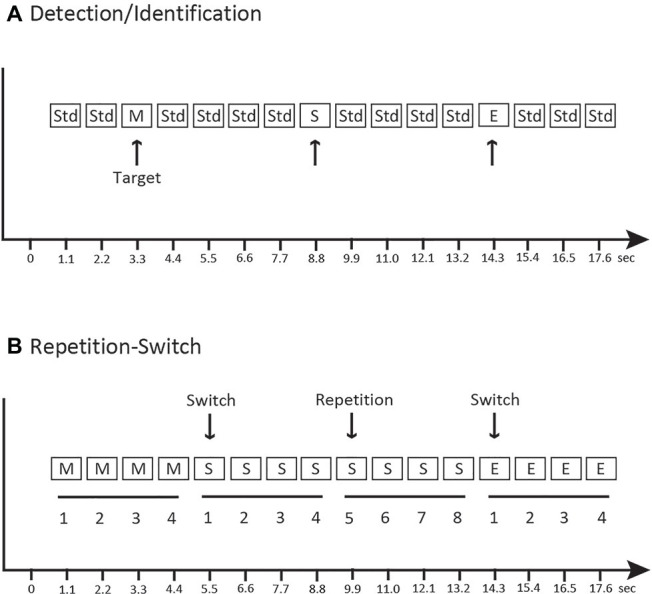
Schematic of the experimental paradigm. **(A)** *Detection* and *Identification* conditions: music (M), speech (S), and environmental (E) sounds were randomly dispersed with a complex tone (1,000 Hz, “Std”). The target sounds are indicated with an arrow and were the same sounds in both conditions, with the difference being whether the key was pressed when any sound was detected vs. pressing a specific key to identify the category of sound. **(B)** *Repetition-Switch* condition: categories of music, speech, and environmental sounds were presented randomly, in groups of four repetitions. The arrow indicates a switch of category and a repeat of category. The *x*-axis indicates time.

Participants sat in a comfortable chair in an electrically shielded and sound-controlled booth (IAC Acoustics, Bronx, NY). Stimuli were presented with two speakers placed approximately 1.5 m, 45° to the left of center and 1.5 m, 45° to the right of center from the seated listener. Sounds were presented in three conditions (*Detection, Identification, and Repetition-Switch*). Figure 2 provides a schematic of the experimental conditions. All 22 participants completed all tasks. In the *Detection* condition (Figure 2A), the categorical sounds from the three categories (speech, music, and environmental) were randomly interspersed with the 1,000 Hz tone (Std) in an auditory oddball paradigm. Sounds were presented in a quasi-randomized order such that no two novel sounds occurred in succession. Participants performed a go/no-go task in the *Detection* condition – listen to the sounds and press a single response button as soon any novel sound was detected; withhold a button press for all of the standard (1,000 Hz) tones. Only detection of the categorical sounds was required. In the *Identification* condition (Figure 2A), the same oddball stimuli were presented as in the *Detection* condition (in differently randomized oddball sequences). The participant’s task in the *Identification* condition was to listen to the sounds and press a specific button uniquely corresponding to each category of novel sounds (speech, music, or environmental) but withhold a button press for all of the standard (1,000 Hz) tones. In this condition, detection of the novel sound among the complex tones and then identification of the target stimulus was required. For the *Detection* and *Identification* conditions, 240 stimuli were presented in four differently randomized blocks with a ratio of 0.2 novel sounds and 0.8 standard sounds (960 tones in total, 192 standards and 48 deviants, 16 of each category type). Novel tokens were not repeated within a stimulus block in the *Detection* and *Identification* conditions.

In the *Repetition-Switch* condition, only the novel speech, music, and environmental sounds were presented (no 1,000 Hz tones). Sounds were repeated in groups of four: when a category of sound was presented, four distinct tokens from that category were individually presented and then switched to another sound category (Figure 2B). Presentation of sounds was quasi-randomized such that categories could only repeat successively one time. Thus, sounds occurred in groups of either four or eight repetitions in each category. This was a three-alternative forced-choice task. The participant’s task was to listen to and classify each sound by pressing the button that uniquely corresponded to the sound category (speech, music, or environmental). Participants were not told anything about the patterned structure of the stimulus sequence only that they were to decide which category the sound belonged to on each trial. For the *Repetition-Switch* condition, 240 stimuli were presented in each of six blocks with a ratio of 0.33 speech sounds, 0.33 music sounds, and 0.33 environmental sounds (1,440 stimuli in total, 480 speech, 480 music, and 480 environmental).

A within-subjects design was used so that all participants performed all of the three conditions. The order of conditions was randomized across participants in a Latin square design. Participants were instructed about the task before each condition and were provided with a short practice to make sure they understood their task. Total session time, including instructions, practice, testing, and breaks, was approximately 1.25 h.

### Data Analysis

This report includes data from all of the 22 participants of the study in the three conditions conducted (*Detection, Identification and Repetition-Switch*). There were no exclusions. Hit rate (HR) and reaction time (RT) were calculated for the responses to the novel sounds, separately, in each condition. Hits were calculated as button presses that occurred 100–1,100 ms from tone onset in the *Detection* condition, and 100–1,800 ms in the *Identification* condition. Misses were calculated as no response to novel sounds within the designated time interval. False alarms were calculated as a button press to a standard sound in the *Detection* condition and were calculated as a misidentification in the *Identification* condition (e.g., pressing the key for speech or environment when a music stimulus occurred). In the *Repetition-Switch* condition, HR and RT were calculated for each category (using a 100–1,100 ms window), and separately for each position within the group of four or eight. The mean was based on calculating the HR or RT to all of the speech sounds that occurred in Position 1 separately from all speech sounds that occurred in Position 2 and so on, for each sound category. False alarms were misidentification of the category.

### Statistical Analyses

A two-way repeated measures ANOVA was used to assess and compare HR and RT, separately, to novel sounds in the *Detection* and *Identification* conditions. In cases where data violated the assumption of sphericity, the Greenhouse-Geisser estimates of sphericity were used to correct the degrees of freedom. Corrected values of *p* are reported. Tukey’s HSD for repeated measures was conducted on pairwise contrasts for *post hoc* analyses when the omnibus ANOVA was significant. Contrasts were reported as significantly different at *p* < 0.05. Statistical analyses were performed using Statistica 13.3 software (Tibco).

## Results

### Reaction Time to Novel Sounds

**Figure 3:**
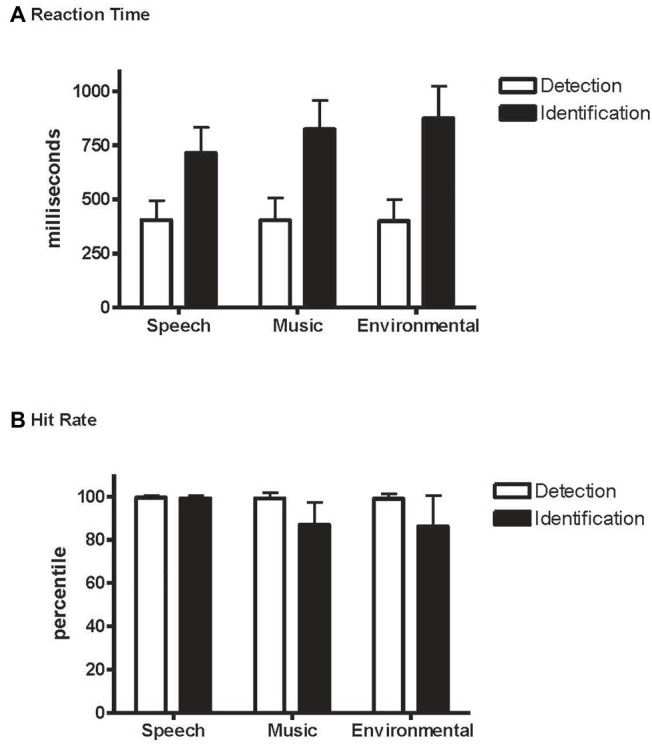
*Detection* and *Identification* conditions. **(A)** Reaction time (RT): mean reaction time to speech, music, and environmental sounds, indicated separately with white bars for the *Detection* condition and with black bars for the *Identification* condition. Whiskers show the standard deviations. **(B)** Hit rate (HR): mean hit rate to speech, music, and environmental sounds, indicated separately with white bars for the *Detection* condition and black bars for the *Identification* condition. Whiskers show the standard deviations.

Overall, it took longer to identify the novel sounds (804 ms) than to detect them (403 ms) (main effect of condition, *F*_1,21_ = 315.7, *p* < 0.0001, $\eta_{p}^{2}$ = 0.94; Figure 3A), and RT was fastest to the speech sounds (*F*_2,42_ = 43.3, *ε* = 0.99, *p* < 0.0001, $\eta_{p}^{2}$ = 0.67). However, an interaction between condition and stimulus type revealed an effect of RT to speech but only when identifying the novel sounds, not when detecting them as novel among the standards without identifying them (condition × stimulus type interaction, *F*_2,42_ = 49.2, *ε* = 0.95, *p* < 0.0001, $\eta_{p}^{2}$ = 0.70). RT did not differ by category when pressing for any detected novel sound (Table 1 and Figure 3A, white bars), whereas response times were fastest to speech when identifying the novel sounds (Table 2 and Figure 3A, black bars).

**Table 1:** Detection condition.

	Mean hit rate	Mean reaction time (ms)
Speech	0.99 (0.009)	405 (90)
Music	0.99 (0.02)	404 (102)
Environmental	0.99 (0.02)	400 (99)

Standard deviation is in parentheses.

**Table 2:** Identification condition.

	Speech	Music	Environment
Hit rate			
Speech	**0.99 (0.01)**	0.007 (0.01)	0.0009 (0.003)
Music	0.009 (0.02)	**0.87 (0.10)**	0.09 (0.09)
Environment	0.007 (0.009)	0.07 (0.07)	**0.86 (0.14)**
Reaction time (s)			
Speech	**0.714 (0.119)**	0.661 (0.077)	1.010 (0)
Music	0.895 (0.495)	**0.824 (0.135)**	1.025 (0.284)
Environment	0.726 (0.187)	0.914 (0.244)	**0.875 (0.148)**

Standard deviation is in parentheses. Target responses are depicted in bold. Misidentifications are depicted in regular type.

### Accuracy for Detecting and Identifying Novel Sounds

Overall, responses were more accurate when detecting a novel sound (0.99) than when identifying one (0.91) (main effect of condition on hit rate, *F*_1,21_ = 45.2, *p* < 0.0001, $\eta_{p}^{2}$ = 0.68). Responses to speech stimuli were more accurate than to music or environmental sounds with no difference in HR between music and environmental sounds (main effect of stimulus type in HR, *F*_2,42_ = 12.9, *ε* = 0.82, *p* < 0.0001, $\eta_{p}^{2}$ = 0.38; Figure 3B). However, the interaction between condition and stimulus type (*F*_2,42_ = 12.10, *ε* = 0.78, *p* < 0.0001, $\eta_{p}^{2}$ = 0.37) showed that HR was higher for speech than music and environmental stimuli only in the *Identification* condition. That is, the speech effect occurred only when the task involved both detecting and identifying the category of novel sounds (*Identification* condition), and not when only detecting that there were novel sounds in the sequence (*Detection* condition). False alarm responses were mainly due to misidentification errors and occurred mainly to the music and environmental sounds in the *Identification* condition (Table 2).

### Reaction Time for Switch and Repeat of Sound Categories

**Figure 4:**
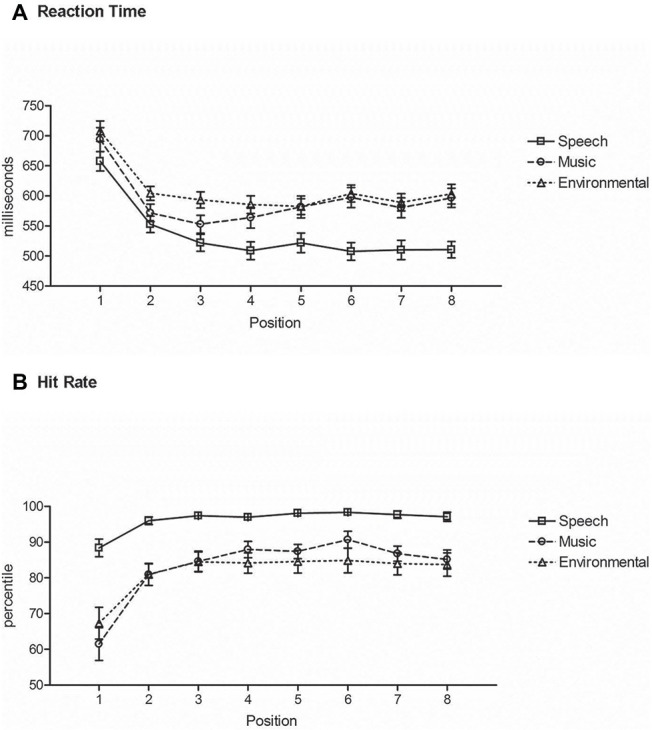
*Repetition-Switch* condition. **(A)** Reaction time: mean RT (in milliseconds, *y*-axis) at each position (*x*-axis) for responses to speech (square, solid line), music (circle, dashed line), and environmental (triangle, dotted line) sounds. Position 1 is a category switch and Position 5 is a category repeat. **(B)** Hit rate: mean HR (*y*-axis) at each position (*x*-axis) for responses to speech (square, solid line), music (circle, dashed line), and environmental (triangle, dotted line) sounds. Position 1 is a category switch and Position 5 is a category repeat. Whiskers show the standard deviations.

RT to speech sounds was shorter than to any other category of sounds (main effect of stimulus type, *F*_2,42_ = 55.7, *ε* = 0.99, *p* < 0.0001, $\eta_{p}^{2}$ = 0.73) (Figure 4A). *Post hoc* calculations showed that there was no difference in RT between music and environmental sounds. RT was longest at the category switch position (Position 1), with a decrease in RT after a single repetition (Position 2) for all categories (main effect of position, *F*_7,147_ = 65.3, *ε* = 0.35, *p* < 0.0001, $\eta_{p}^{2}$ = 0.76). RT decreased again in Position 3 but only for speech sounds (interaction between stimulus type and position, *F*_14,294_ = 5.8, *ε* = 0.46, *p* < 0.0001, $\eta_{p}^{2}$ = 0.22). RT at Position 5 of a repeated category, where a category switch could have occurred, did not differ from the RT at Position 4 where no switch could have been anticipated (Figure 4A).

### Accuracy for Classifying Sound Categories That Switch and Repeat

Hit rate was highest for speech sounds than other sounds (main effect of stimulus type, *F*_2,42_ = 25.3, *ε* = 0.90, *p* < 0.0001, $\eta_{p}^{2}$ = 0.54), with no difference in HR between music and environmental sounds (Figure 4B). The switch position (Position 1) had a lower hit rate than any other position (main effect of position, *F*_7,147_ = 35.1, *ε* = 0.22, *p* < 0.0001, $\eta_{p}^{2}$ = 0.63), and no difference in HR with any other positions. The interaction between stimulus type and position (*F*_14,294_ = 8.85, *ε* = 0.46, *p* < 0.0001, $\eta_{p}^{2}$ = 0.30) was due to Position 2 for music also being lower than Positions 3–7, where this did not occur for speech or environmental sounds (Position 2 was not different from any of the other positions in those series) (Figure 4B).

### Comparison Across Task Conditions

**Figure 5:**
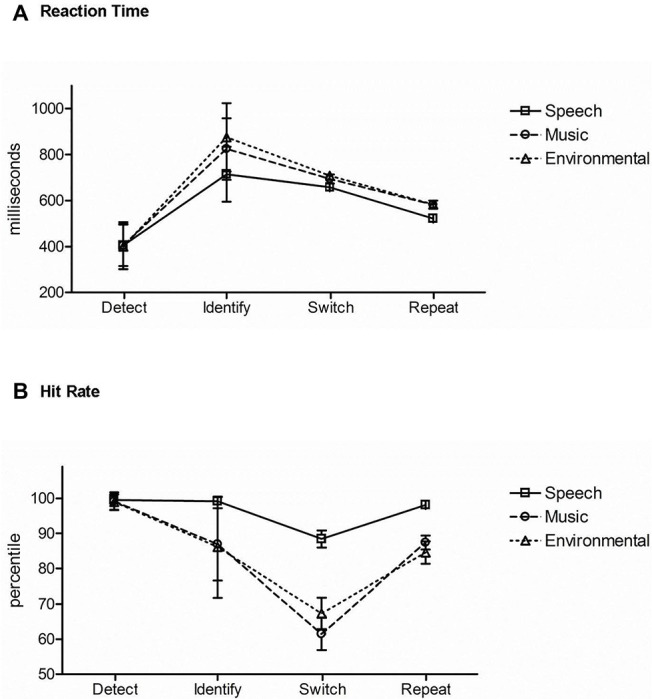
Comparison of task responses across conditions. **(A)** Reaction time: mean RT is displayed in response to speech (square, solid line), music (circle, dashed line), and environmental (triangle, dotted line) sounds for each task (denoted across the *x* axis): the *Detection* condition (Detect), *Identification* condition (Identify), and the *Repetition-Switch* condition with Position 1 switch trials (Switch), and Position 5 repeat trials (Repeat). **(B)** Hit rate: mean HR is displayed for speech (square, solid line), music (circle, dashed line), and environmental (triangle, dotted line) sounds for each task (denoted across the *x* axis): the *Detection* condition (Detect), *Identification* condition (Identify), and the *Repetition-Switch* condition with Position 1 switch trials (Switch), and Position 5 repeat trials (Repeat). Whiskers show standard deviations.

Figure 5 displays the results across the three task conditions for comparison. *Reaction time*: there was a main effect of task condition, (*F*_2,42_ = 160.96, *ε* = 0.99, *p* < 0.0001, $\;\eta_{p}^{2}$ = 0.88). *Post hoc* analyses showed that RT was fastest when detecting a novel sound without identifying it (Figure 5A, *Detect*). When recognizing and categorizing sounds, RT was faster when the category of sound was expected (Figure 5A, *Switch* and *Repeat*) than when the category was unexpected (Figure 5A, *Identify*). There was also a main effect of stimulus category (*F*_2,42_ = 43.71, *ε* = 0.97, *p* < 0.0001, $\;\eta_{p}^{2}$ = 0.68). Mean RT to speech tokens was fastest (Figure 5A, solid line) and mean RT to environmental sounds slowest (Figure 5A, dotted line). There was an interaction between task condition and stimulus category (*F*_4,84_ = 36.19, *ε* = 0.69, *p* < 0.0001, $\;\eta_{p}^{2}$ = 0.63). *Post hoc* calculations showed that the effect of stimulus category was due to classification of the stimulus in the *Identification* and *Repetition-Switch* conditions, in which responses to speech were faster and more accurate. That is, stimulus category had no effect on RT when the task was to simply recognize any unexpected novel stimulus among complex tones (*Detection* condition).

*Hit rate*: there was a main effect of stimulus category (*F*_2,42_ = 47.24, *ε* = 0.93, *p* < 0.0001, $\;\eta_{p}^{2}$ = 0.69). *Post hoc* calculations showed that mean hit rate was highest for speech sounds (*p* < 0.0001) (Figure 5B, solid line) over music and environmental sounds (*p* = 0.52) (Figure 5B, dashed and dotted lines). There was also a main effect of task condition (*F*_2,42_ = 46.56, *ε* = 0.61, *p* < 0.0001, $\;\eta_{p}^{2}$ = 0.69). *Post hoc* calculations showed that mean HR was highest in the *Detection* condition (Figure 5B, *Detect*) and lowest for the switch trials in the *Repetition-Switch* condition (Figure 5B, *Switch*). An interaction between task condition and stimulus category was due to speech having the highest mean HR when the task involved categorizing the sounds: in the *Identification* condition (Figure 5B, *Identify*) and in the *Repetition-Switch* condition (Figure 5B, *Switch*, *Repeat*), whereas there was no difference in mean HR between music and environmental sounds. There was no effect of stimulus category on HR in the *Detection* condition (Figure 5B, *Detect*).

## Discussion

Three conditions were conducted to test processing differences by stimulus category (speech, music, and environmental) when detecting and identifying unexpected and expected sounds. Response time and accuracy were modulated by the processes required to perform the specific task (e.g., detection and identification) and were modulated by the expectation of a sound category. Sound category itself had no effect on sound detection outcomes – pressing the response key to any novel sound in the sequence. In contrast, the sound category modulated the HR and RT when the task required identification, both when the sounds were unexpected and when the sound category was expected.

Sound category had no effect on accuracy or reaction time for sound detection. That is, in the common auditory oddball paradigm in which deviant tones occur among standard tones and the task requires that the listener detect the unexpected occurrence of novel (deviant) tokens within a sequence of standard tones (*Detection* condition), speech sounds showed no advantage over other categorical sounds. In contrast, when the task required detection and identification of the category of unexpected novel sounds in the auditory oddball sequence (*Identification* condition), sound category had a dramatic impact on both accuracy and reaction time. Speech sounds were classified faster (RT was shorter) and more accurately (HR was higher and error rate lower) than non-speech sounds (music and environmental). Furthermore, it took 300 ms longer to identify speech sounds than to detect them, and 420–475 ms longer to identify non-speech sounds than to detect them. For speech sounds, accuracy did not differ between detecting and identifying them, but the response speed was faster to detect than identify (Figure 3, compare black and white bars for speech in A and B). In contrast, categorizing the music and environmental sounds decreased accuracy by 12% compared to detecting them (Figure 3B, compare black and white bars for music and environment). These results are consistent with Agus et al. (2012) who assessed the response speed to sung vocal sounds compared to instrumental sounds. The Agus et al. study compared a go/no-go task, in which half the sounds were vocal and half were music sounds (go for speech/no-go for music) to a simple detection task (pressing a response key to all of the sounds in the sequence). They found a time advantage for voice recognition that was on average 105 ms faster to speech compared to music, and similarly as was found in the current study, RT was faster for detection compared to identification of a sound category. Agus et al. (2010, 2012) interpreted the voice advantage as being due to speech being processed differently than music as opposed to some consequence of spectro-temporal features of the speech sounds themselves. Faster and more accurate processing of speech sounds could be attributed to speech being specialized for processing (Eimas, 1974; Pisoni, 1979; Liberman and Mattingly, 1989; Belin et al., 2000; Parviainen et al., 2005; Uppenkamp et al., 2006; Vouloumanos and Werker, 2007; Norman-Haignere et al., 2015) with different brain areas responsible for recognition of music sounds (Norman-Haignere et al., 2015) than for voice recognition (Belin et al., 2000; Chartrand et al., 2008). Thus, it is possible that the neural circuitry controlling speech sounds enable a more rapid and reliable pattern for the correct selection.

In the *Repetition-switch* condition, the task required every sound in the sequence to be detected and identified as speech, music, or environmental. RT was shorter and accuracy was higher to speech sounds overall than to non-speech sounds. This pattern of results was similar to those in the *Identification* condition that required detecting and identifying unexpected novel sounds. However, when the sequence contained only categorical sounds (in the *Repetition-switch* condition), RT was faster for switch and repeat trials compared to responses to the same sounds in the *Identification* condition in which the task involved detecting and identifying unexpected novel sounds among non-categorical, complex sounds (Table 3 and Figure 5, compare *Identification* with *Switch/Repeat* conditions, both required classification of the sounds). This result suggests that identifying the novel sounds among non-categorical sounds involves additional processing time prior to categorizing the sounds. The reason is that the novel sounds first have to be detected as novel and then classified to the category they belong in the *Identification* condition, whereas in the *Repetition-Switch* condition, the detection step is not required to perform the task. Each sound could be expected to be one of the three sound categories. Thus, with only the process of categorization for each sound, the mean response time was faster.

**Table 3:** Comparison of responses across conditions.

	Reaction time (mean/SD)	Hit rate (mean/SD)
Detect Speech	405 (90)	99 (1)
Identify Speech	714 (119)	99 (1)
Switch Speech 1	658 (74)	88 (11)
Repeat Speech 4	509 (69)	97 (4)
Repeat Speech 5	522 (77)	98 (3)
Detect Music	404 (102)	99 (3)
Identify Music	824 (134)	87 (10)
Switch Music 1	694 (87)	61 (21)
Repeat Music 4	564 (82)	88 (11)
Repeat Music 5	582 (85)	87 (9)
Detect Env	400 (99)	99 (2)
Identify Env	875 (148)	86 (14)
Switch Env 1	708 (76)	67 (20)
Repeat Env 4	586 (69)	84 (13)
Repeat Env 5	582 (62)	85 (15)

Env, environment; for the *Repetition-Switch* condition, Position 1 stimuli, 1; Position 4 stimuli, 4; Position 5 stimuli, 5.

There was also a response difference for the switch and repeat trials that was influenced by the stimulus category. In the *Repetition-switch* condition, RT was longer for switch than for repeat trials. There was a dramatic decrease in RT at the first repeat trial of a sound category (∆100 ms, Figure 4A). RT continued to decrease on repeat trials for speech sounds but did not decrease further after the first repeat for the non-speech sound categories. We further predicted that mean RT for repeat trials would be similar to the mean RT in the *Detection* condition (~400 ms) because once the category was categorized at the switch trial, the repeat trials would only need to be detected. That is, the categorization step would have already been confirmed implicitly. However, that was not the case. Mean RT for the repeat trials was on average 100 ms slower for speech tokens than simple detection of speech in the *Detection* condition, and more than 160 ms slower for music and environmental sounds than recognition of the same sounds (Table 3).

There was also a dramatic change in HR from the switch to the repeat trials. Hit rate plateaued very rapidly, at the first repetition of a sound category (Figure 4B). We expected that if participants implicitly learned the global patterned structure of the sounds (tokens within a sound category repeated four or eight times) then HR would increase to 100% at the first repeat trial after a switch trial because the category of sound would be known for Positions 2–4. However, this occurred only for the speech category, in which HR reached 99% accuracy from the first repeat trial (up from 88% at the switch trial). For the non-speech categories, only 85–88% accuracy was achieved for the repeat trials (up from 61 to 65% at the switch trial). These results suggest that participants were making judgments on every sound in the *Repetition-Switch* condition, even during the repeat trials and were not relying fully on the knowledge of category repetition that could have been inferred from the global structure of the sound sequence. That is, once the switch occurred, accurate responses would have only required sound detection for repeat trials. The question then was why. In part, this result may be explained by the experimental design in which no two of the same stimulus tokens were repeated within a category, only the category was repeated from trial to trial. For example, a telephone ringing, bird chirping, and car revving would all require the same categorical response (environmental). Thus, we reason that some level of judgment occurred for each repeat stimulus presentation to confirm that the stimulus token indeed belonged to the same category, even when implicitly knowing that the category was repeated. In this light, the speech recognition advantage is even more striking because any additional processing to confirm category-fitting did not alter response times or accuracy for speech.

Having expected that the global pattern of the stimulus sequences would be implicitly learned, we also predicted that this would have an effect on processing of the Position 5 stimulus (Figure 4). Most of the time there was a switch in category after four repeats (two-thirds of the time), and thus it could have been implicitly anticipated. That is, participants would have expected a switch more often than a repeat if they had implicitly detected the global pattern. We, therefore, predicted that there would be a longer RT for Position 5 stimuli when the category did not switch, reflecting some expectation of a category switch (i.e., the prediction was not met). However, there was no difference in mean RT and HR between Position 4 where no switch could have been anticipated and Position 5 where a switch could be anticipated. This result could also be explained as an effect of there being no repeated stimuli within a category and classification of the token had to be confirmed on every trial because only the category repeated but not the sound tokens within the category. Another possible interpretation is that a strong expectation was not built up implicitly, or that the global pattern of the stimuli was not implicitly learned. In this case, participants may have been operating on a local basis – classifying every token as it occurred, irrespective of any global structure within the sequence and thus “switch costs” were observed only when a category actually changed.

The “switch cost” in the *Repetition-Switch* condition was calculated as the difference between the last repeat trial (Position 4) before the switch trial (Position 1). For speech this would be ∆149 ms (509 vs. 658 ms), for music ∆130 ms (564 vs. 694 ms), and for environmental sounds ∆122 ms (582 vs. 708 ms). Thus, there was a larger switch cost for speech than for non-speech sounds because RT decreased more, on average, for the repeated speech trials. It is interesting to note that overall, RT was faster for the switch trials in the *Repetition-Switch* condition where there was an RT “cost” than for the categorization trials of the same tokens in the *Identification* condition where there was no task “cost” (Figure 5). This suggests that there was additional processing when classifying sounds among non-categorical sounds (detect and identify) than for categorizing among different types of sounds. The novel tones must first be detected among the non-categorical complex sounds and identified as belonging to a particular category. This took longer than classifying them when all the sounds were categorical and the task was to sort them according to their classification.

## Conclusions

Faster and more accurate responses to speech were found only when classifying the category of sounds. When the task was to respond to randomly occurring real-life sounds among non-categorical tones in a go/no-go oddball paradigm, detection of a novel sound showed no sound category advantage and no speech preference. There was also a greater advantage for speech perception (faster RT and higher accuracy) over other sounds when the category of sound could be anticipated. The current results thus indicate that while speech is special, its advantage in processing is due to the task requirements and to a level of processing that involves categorization of sounds in the environment. There was a faster and more accurate response profile for speech sounds overall, both when detecting and identifying sounds among non-categorical sounds (categories were unexpected) and when detecting and identifying among only categorical sounds (categories were expected). Thus, our results overall are consistent with a theory of speech processing relying on specialized systems over music and other environmental sounds (McQueen et al., 2019).

## Data Availability Statement

The raw data supporting the conclusions of this article will be made available by the authors, without undue reservation.

## Ethics Statement

The studies involving human participants were reviewed and approved by Albert Einstein College of Medicine Institutional Review Board. The patients/participants provided their written informed consent to participate in this study.

## Author Contributions

ES and HM designed the experiment. WL collected the data and assisted with data analysis and figure preparation. ES and HM analyzed and interpreted the data. ES and HM prepared the figures and wrote the manuscript.

## Conflict of Interest

The authors declare that the research was conducted in the absence of any commercial or financial relationships that could be construed as a potential conflict of interest.
